# N-terminally truncated Aβ4-x proteoforms and their relevance for Alzheimer’s pathophysiology

**DOI:** 10.1186/s40035-022-00303-3

**Published:** 2022-06-01

**Authors:** Agueda Rostagno, Erwin Cabrera, Tammaryn Lashley, Jorge Ghiso

**Affiliations:** 1 Departments of Pathology, New York University School of Medicine, New York, NY 10016 USA; 2 The Queen Square Brain Bank for Neurological Disorders, Department of Clinical and Movement Neurosciences, UCL Queen Square Institute of Neurology, London, WC1N 3BG UK; 3 Department of Neurodegenerative Disease, UCL Queen Square Institute of Neurology, London, WC1N 3BG UK; 4 Departments of Psychiatry, New York University School of Medicine, 550 First Avenue, New York, NY 10016 USA; 5 Current affiliation: Farmingdale State College, State University of New York, Farmingdale, NY 11735 USA

**Keywords:** Alzheimer’s disease, Amyloid-β truncated species, Peptide oligomerization, Brain clearance, Brain efflux, Stereotaxic intracerebral injection

## Abstract

**Background:**

The molecular heterogeneity of Alzheimer’s amyloid-β (Aβ) deposits extends well beyond the classic Aβ1-40/Aβ1-42 dichotomy, substantially expanded by multiple post-translational modifications that increase the proteome diversity. Numerous truncated fragments consistently populate the brain Aβ peptidome, and their homeostatic regulation and potential contribution to disease pathogenesis are largely unknown. Aβ4-x peptides have been reported as major components of plaque cores and the limited studies available indicate their relative abundance in Alzheimer’s disease (AD).

**Methods:**

Immunohistochemistry was used to assess the topographic distribution of Aβ4-x species in well-characterized AD cases using custom-generated monoclonal antibody 18H6—specific for Aβ4-x species and blind for full-length Aβ1-40/Aβ1-42—in conjunction with thioflavin-S and antibodies recognizing Aβx-40 and Aβx-42 proteoforms. Circular dichroism, thioflavin-T binding, and electron microscopy evaluated the biophysical and aggregation/oligomerization properties of full-length and truncated synthetic homologues, whereas stereotaxic intracerebral injections of monomeric and oligomeric radiolabeled homologues in wild-type mice were used to evaluate their brain clearance characteristics.

**Results:**

All types of amyloid deposits contained the probed Aβ epitopes, albeit expressed in different proportions. Aβ4-x species showed preferential localization within thioflavin-S-positive cerebral amyloid angiopathy and cored plaques, strongly suggesting poor clearance characteristics and consistent with the reduced solubility and enhanced oligomerization of their synthetic homologues. In vivo clearance studies demonstrated a fast brain efflux of N-terminally truncated and full-length monomeric forms whereas their oligomeric counterparts—particularly of Aβ4-40 and Aβ4-42—consistently exhibited enhanced brain retention.

**Conclusions:**

The persistence of aggregation-prone Aβ4-x proteoforms likely contributes to the process of amyloid formation, self-perpetuating the amyloidogenic loop and exacerbating amyloid-mediated pathogenic pathways.

## Background

Alzheimer’s disease (AD), the most common type of dementia, is neuropathogically characterized by the presence of hyperphosphorylated tau in intraneuronal neurofibrillary tangles, the deposition of amyloid-β (Aβ) in the brain parenchyma and cerebral vasculature, and the gradual loss of synapses which is the best pathological correlate with cognitive impairment [1, 2]. Although it remains unclear what primarily triggers and drives the progression of AD, different lines of investigation point out to a central role of Aβ in the disease pathogenesis and support the relevance of oligomeric conformations of the peptide [1, 3–6]. The transition from soluble monomeric species normally present in body fluids to the oligomeric, protofibrillar and endpoint fibrillar assemblies is considered today a significant contributor to the disease pathobiology. Intermediate oligomeric and protofibrillar forms have been shown to display the most potent effects in neuronal cells inducing synaptic disruption, neurotoxicity, and ultimately neurodegenerative cell death [4, 7].

Recent evidence indicates that the molecular heterogeneity of Aβ deposits is significantly more complex than originally anticipated, extending well beyond the Aβ1-40/Aβ1-42 dichotomy and being substantially expanded by the presence of multiple post-translational modifications that increase the proteome diversity [8]. Among the protein modifications reported for Aβ, one of the most studied as species likely involved in initial nucleation or seeding events is the cyclation of N-terminal glutamate to form pyroglutamate (pE) in truncated forms of Aβ starting at Glu3 and Glu11 [9]. The loss of a negative charge resulting from this cyclation dramatically changes the peptide structure, increases the β-sheet content, and alters the hydrophobicity of the molecule, enhancing the aggregation propensity and resistance to enzymatic degradation of the post-translationally modified Aβ forms [10, 11]. These structural changes support a contribution of pE-modified species to the disease process as suggested by their exacerbated neurotoxicity [12–15] and their abundance in AD brains compared to cognitively intact age-matched controls [16–20]. Additional N- and C-terminal truncations that significantly contribute to the heterogeneity and complexity of the Aβ profile in biological fluids and brain deposits have been increasingly identified in recent years [21, 22]. These multiple degradation products are likely generated by the action of a number of Aβ-degrading enzymes, among which are neprilysin, insulin-degrading and endothelin-converting enzymes, plasmin, ADAMTS4 (a disintegrin and metalloproteinase with thrombospondin motifs 4), and matrix metalloproteases [23–29]. In addition to the classic Aβ peptides generated from the precursor protein APP by the combined activity of BACE1 and γ-secretase—starting at the aspartate residue at position 1 and ending at amino acids 38/40/42—multiple truncated Aβ species exhibiting different solubility properties have been identified in cellular and animal models as well as in AD patients [11, 30–40]. C-terminally cleaved fragments—among which is Aβ1-34, a fragment generated by the action of the matrix metalloproteases MMP-2 and MMP-9 [27] as well as by BACE1 cleavage [41]—have been described as components of the heterogeneous cerebrospinal fluid (CSF) Aβ profile [31, 42, 43] and as constituents of soluble extracts obtained from amyloid brain deposits in AD and various Tg models [38, 39], suggesting their role in clearance mechanisms. In addition to the pE-modified forms described above, additional N-terminally truncated Aβ peptides have also been reported, including Aβ2-x, Aβ4-x, Aβ5-x, and Aβ17-x [39, 44, 45]. Among these N-terminal truncations, species beginning at Phe4 and bearing an intact C-terminus are especially relevant due to their poor solubility properties [39]. Indeed, Aβ4-42 peptides were reported more than 3 decades ago as major components of amyloid plaque cores [46] and the limited studies available seem to indicate their relative abundance in patients with AD, Down’s syndrome, and vascular dementia [39, 40, 45, 47–51].

The mechanisms leading to the development and progression of AD are complex and likely involve different cellular pathways. Emerging data indicate that the reduced brain Aβ clearance—particularly relevant in the elderly—plays a critical role in amyloid formation and AD pathogenesis [52]. Work from our group and others has shown that Aβ efflux from brain interstitium is a fast process that follows local and systemic paths and that, in addition to the blood–brain barrier (BBB), local enzymatic degradation and the bulk flow transport through the choroid plexus into the CSF play significant roles [39, 53–58]. Notably, most of the knowledge in the field is based on experimentation performed primarily with monomeric Aβ1-40 or with poorly characterized synthetic homologues. Our recent in vivo findings [38, 59, 60] draw attention to major clearance kinetic differences for oligomeric forms of Aβ1-40, highlighting a differential brain retention of oligomers compared to monomeric counterparts. Currently, no information is available on brain efflux of the truncated Aβ forms and how their oligomerization, particularly in the case of Aβ4-x species exhibiting high aggregation propensity, poor solubility, and relative high abundance in AD deposits, impacts brain removal, favoring their accumulation and tissue deposition. The present work was designed to address this gap in knowledge and provide a quantitative evaluation of the differential brain removal efficiency of pathogenic oligomeric Aβ species, thereby contributing to a better understanding of the delicate balance between effective clearance and formation of Aβ deposits.

## Methods

### Human brain tissue samples

Post-mortem human brains were from Queen Square Brain Bank (QSBB) for Neurological Disorders (UCL Queen Square Institute of Neurology). All tissue samples were donated with the full, informed consent. Accompanying clinical and demographic data of all cases used in this study were stored electronically in compliance with the 1998 data protection act. Ethical approval for this study was obtained from the NHS research ethics committee (NEC) and in accordance with the human tissue authority’s (HTA’s) code of practice and standards under license number 12198. All cases were diagnosed pathologically according to current consensus criteria [61–65]. The standard diagnostic criteria for the neuropathological diagnosis of AD and the presence of cerebral amyloid angiopathy (CAA) were used in all cases. Sequential formalin-fixed paraffin-embedded sections from the frontal cortex of the AD cases are summarized in Table 1.

**Table 1:** Case demographics of post-mortem human cases used in immunohistochemical analyses

Case	PMI (h)	AAO (years)	AAD (years)	Duration (years)	Gender	Diagnosis	Brain weight (g)	APOE	Braak Tau	Thal phase	CERAD	ABC	CAA
1	115:35	49	54	5	M	AD	910	2/3	6	5	3	A3B3C3	3
2	33:26	63	74	11	M	AD	1022	4/4	6	5	3	A3B3C3	3
3	75:20	59	75	16	F	AD	1057	3/4	6	5	3	A3B3C3	3
4	58:50	43	58	15	F	AD	1075	3/3	6	5	2	A3B3C2	3
5	85:35	72	88	16	M	AD	1120	3/4	6	5	3	A3B3C3	1
6	58:10	72	88	16	M	AD	1084	2/3	6	5	2	A3B3C2	3
7	134:20	70	79	9	M	AD	1425	3/4	6	5	3	A3B3C3	3
8	60:00	62	76	14	F	AD	1020	3/4	6	5	3	A3B3C3	3
9	50:35	79	88	9	F	AD	944	3/4	6	5	3	A3B3C3	3
10	90:20	70	86	16	F	AD	1065	3/4	6	5	3	A3B3C3	3
11	40:10	na	77	na	M	Control	1327	2/2	0	0	0	A0B0C0	2
12	29:30	na	78	na	F	Control	1225	2/2	2	0	0	A0B2C0	0
13	38:50	na	71	na	M	Control	1480	3/3	1	0	0	A0B1C0	0

Cases were pathologically diagnosed with AD based on the current diagnostic criteria. Control cases were selected based on the absence of any neurological disorder.

*PMI* post-mortem interval recorded in hours; *AAO* age at disease onset; *AAD* age at death; *CAA* cerebral amyloid angiopathy; *F* female; *M* male

### Immunohistochemistry

#### Single immunohistochemical analysis

Slides with 8-µm mounted tissue sections were incubated at 60 °C overnight, followed by deparaffinization in xylene, rehydration in decreasing grades of alcohol, and subsequent incubation in methanol/H_2_O_2_ (0.3%) solution for 10 min to block endogenous peroxidase activity. After 10-min pretreatment in 100% formic acid, sections were subjected to heat-induced antigen retrieval in 0.1 M citrate buffer (pH 6.0) and pressure cooked 10 min at maximum pressure. Following incubation in 10% non-fat milk (30 min, room temperature) to block non-specific binding, sections were immunoreacted with the different primary antibodies for 1 h at room temperature. These primary antibodies were the pan Aβ antibody Aβ8-17 (mouse monoclonal Dako M0872, Carpinteria, CA; 1:100), antibody for Aβx-40 (rabbit polyclonal Thermo Fisher 44-348A, Waltham, MA; 1:100, recognizing Aβ forms ending at position 40 and lacking cross reactivity with Aβ1-42), antibody for Aβx-42 (rabbit polyclonal Thermo Fisher 44-344; 1:100, recognizing Aβ forms ending at position 42 and showing no cross reactivity with Aβ1-40), and antibody for Aβ4-x (in-house mouse monoclonal clone 18H6; 1:100) specifically reacting with Aβ species N-terminally truncated at phenylalanine 4 (Phe4) and blind for other N-terminally truncated forms including Aβ2-40/42, Aβ3-40/42, pyroglutamate-modified AβpE3-40/42, Aβ5-40 and Aβ11-40 as well as for the full-length Aβ1-40 and Aβ1-42 [39, 51]. After three gentle 5-min washes in tris-buffered saline containing 0.1% Tween 20 (TBS-T), the slides were incubated for 30 min with either biotinylated goat anti-mouse or biotinylated goat anti-rabbit secondary antibody (Vector Laboratories, Burlingame, CA; 1:200). The tissue sections were subsequently washed as above, incubated in ABC (Dako, Carpinteria, CA) for signal amplification using 3,3′-diaminobenzidine (DAB) as the chromogen, and counterstained in Mayer’s haematoxylin (VWR, Poole, United Kingdom). Finally, sections were dehydrated in increasing grades of alcohol, cleared in xylene, and mounted. To provide a semi-quantitative assessment, the following scoring criteria were used to characterize the immunostainings encompassing all pathological hallmarks: 0 = no staining, 0.5 = barely detectable staining, 1 = weak staining, 2 = moderate staining, 3 = extensive staining. Heat-map was plotted using GraphPad Prism 9.3.0 (GraphPad, San Diego, CA).

#### Double immunofluorescence analysis

Double immunohistochemical studies were undertaken to visualize the spatial location of the different Aβ peptides with and without Thioflavin staining to highlight the presence of fibrillar amyloid deposits. For double immunohistochemical analysis with Aβ antibodies without Thioflavin staining, tissue sections were dewaxed, pre-treated and blocked as detailed above followed by incubation with a combination of Aβ4-x and either Aβx-40 or Aβx-42 antibodies for 1 h at room temperature. After TBS-T washing, sections were incubated with species-appropriate Alexa Fluor-conjugated secondary antibodies (anti-rabbit Alexa Fluor 488 and anti-mouse Alexa Fluor 568, Invitrogen, Waltham, MA; 1:1000; 2 h, room temperature). When performing the immunohistochemical analysis with additional Thioflavin S staining, sections underwent the same protocol as outlined above, albeit using 70% formic acid pretreatment instead of 100%, as this is the maximal concentration the amyloid stain is capable of withstanding. Following this pretreatment, sections were stained with one Aβ antibody (Aβ4-x, Aβx-40 or Aβx-42), reacted with the pertinent Alexa Fluor conjugate, and subsequently incubated with 0.1% Thioflavin S aqueous solution for 7 min, followed by differentiation in 70% alcohol. To assess colocalization of the Aβ forms truncated at Phe4 with full-length peptides, sections were stained with two Aβ antibodies (either Aβ4-x and Aβx-40 or Aβ4-x and Aβx-42) and the Alexa Fluor 568 and Alexa Fluor 405 conjugates were used together with the Thioflavin stain. Cross-reactivity was assessed by the addition of two control sections stained as above with the individual omission of each primary antibody. Representative fluorescent images were captured using a Leica DM5500B fluorescence microscope and Z-stacks were subjected to a blind 3D deconvolution.

### Peptide synthesis

Synthetic homologues of Aβ1-40 and Aβ1-42, either full-length, N-terminally truncated at Phe4 (Aβ4-40, Aβ4-42), or C-terminally truncated at Leu34 (Aβ1-34), were synthesized using *N-tert-*butyloxycarbonyl chemistry at ERI Amyloid Laboratory (Oxford, CT). Peptides were purified by reverse phase-high performance liquid chromatography on a Vydac C4 column (Western Analytical, Murrieta, CA), molecular masses were corroborated by matrix-assisted laser desorption ionization time-of-flight (MALDI-TOF) mass spectrometry (MS), and concentrations were assessed by amino acid analysis, as previously reported [66]. For MALDI-TOF analysis, peptides were separately solubilized in 0.1% formic acid/50% acetonitrile in water and combined with an equal volume of α-4-hydroxycinnamic acid matrix (Agilent Technologies, Santa Clara, CA) previously solubilized at a concentration of 15 g/l in acetonitrile containing 0.1% trifluoroacetic acid; the resulting mixture was spotted in duplicate onto a Bruker Daltonics MTP 384 massive target T aluminum plate and analyzed in a Bruker Daltonics Autoflex MALDI-TOF mass spectrometer (Bremen, Germany) in linear mode using standard instrument settings at the New York University Mass Spectrometry Core for Neuroscience. External calibration was performed using human adrenocorticotropic hormone peptide 18–39 (average mass = 2465.68 Da) and insulin (average mass = 5733.49 Da). In all cases, MS spectra were processed and analyzed by FlexAnalysis (Bruker Daltonics, Billerica, MA).

### Peptide monomerization and oligomerization

All synthetic homologues were incubated at a concentration of 1 mg/ml in 1,1,1,3,3,3,hexafluoro-isopropanol (HFIP; Sigma Chemical Co., St. Louis, MO) for 4 days, a pretreatment that breaks down β-sheet structures and disrupts hydrophobic forces, leading to monodisperse amyloid subunit preparations [67]. The HFIP-pretreated peptides were lyophilized and subsequently reconstituted to 1 mM in 1% NH_4_OH followed by further dilution in phosphate-buffered saline (PBS) to a final concentration of 50 µM and immediately used for radioiodination and direct intracerebral injection. For the studies involving clearance of Aβ oligomers, the different Aβ peptides—reconstituted in PBS as above—were incubated for 24 h at 37 °C to generate low-molecular-mass oligomers, as we previously described [66], prior to radioiodination, since labeling stably formed Aβ assemblies is known to render labeled preparations with unperturbed structural morphology, preventing the generation of undesired atypical oligomers [68]. The resulting oligomers were immediately used for radiolabeling and intracerebral injections.

### Assessment of peptide structural changes

#### Circular dichroism (CD) spectroscopy

Changes in the secondary structure of the different Aβ peptides induced by the oligomerization procedure were estimated by CD spectroscopy, as previously described [66]. The HFIP-pretreated peptides were solubilized in deionized water as above, followed by further dilution to a final concentration of 50 μM in 10 mM phosphate buffer, pH 7.4, containing 150 mM sodium fluoride. Spectra in the far-ultraviolet (wavelength range: 190–260 nm; band-width 1 nm; intervals 1 nm; scan rate 60 nm/min) were recorded for each peptide immediately after solubilization and following 24-h incubation at 37 ºC in a Jasco J-720 spectropolarimeter (Jasco Inc., Easton, MD) using a 1-mm-path quartz cell. For each sample, 15 consecutive spectra were obtained and averaged, the baseline reading was subtracted, and results were expressed in terms of molar ellipticity (deg × cm^2^/dmol).

#### Thioflavin T binding assay

Aggregation of the different Aβ homologues was also monitored by Thioflavin T binding, following previously described protocols [66, 69]. Briefly, 6 μl aliquots from each peptide aggregation time point were added to 184 μl of 50 mM Tris–HCl buffer, pH 8.5, and 10 µl of 0.1 mM thioflavin T (Sigma). Fluorescence was recorded for 300 s in an LS-50B spectrometer (Perkin Elmer, Waltham, MA) with a slit width of 10 nm and excitation and emission wavelengths of 435 and 490 nm, respectively [70].

#### Electron microscopy

Peptide oligomerization was also monitored by electron microscopy, as previously described [38, 66, 71]. Five microliters of either monomeric or oligomeric preparations of the different Aβ peptides were placed onto carbon-coated 400 mesh Cu/Rh grids (Ted Pella, Inc., Redding, CA) and stained with 1% uranyl acetate in distilled water (Polysciences, Inc., Warrington, PA). The stained grids were examined in a Philips CM-12 transmission electron microscope and photographed with a Gatan (4 k × 2.7 k) digital camera at the Image Core Facility of the Skirball Institute of Biomedical Medicine, NYU School of Medicine, as described [66, 71, 72].

### Peptide radiolabeling

One hundred micrograms of either full-length or truncated Aβ species in both monomeric and oligomeric forms were radiolabeled using 1,3,4,6-tetrachloro-3α-6α-diphenylglycoluril pre-coated tubes (Iodogen, ThermoFisher Scientific/Pierce, Waltham, MA) and Na^125^I (1 mCi, PerkinElmer, Waltham, MA), following the manufacturer’s specifications. All radiolabeling procedures were carried out for 20 min in a Standalone Radioiodine IH-350 hood (Atlantic Nuclear, Rockland, MA). Following removal of free Iodine using a desalting polyacrylamide column (D-salt, cut off 1.8 kDa, Pierce), radioactivity of labeled Aβ was assessed in a Perkin Elmer 1470 automated gamma counter (Perkin Elmer, Waltham, MA). Under these experimental conditions, [^125^I] incorporation rendered specific activities in the range of 3–5 µCi/µg with > 98% TCA precipitable counts [53, 73]. Aggregation status of the [^125^I]-labeled Aβ monomeric and oligomeric species was analyzed by autoradiography following 16.5% SDS–polyacrylamide gel electrophoresis and subsequent direct exposure to HyBlot CL film (Denville Scientific Inc., South Plainfield, NJ), as we previously described [38]. If not immediately used for the clearance experiments, [^125^I]Aβ oligomeric peptides were stored at -80 ºC and used within a week to prevent further oligomerization and minimize radiolysis.

### Intracerebral inoculation of radio-iodinated Aβ peptides

C57BL/6 mice (Taconic Biosciences, Hudson, NY) were intracerebrally injected with the different Aβ preparations following institutionally approved IACUC protocols, essentially as previously reported by our laboratory [38, 74]. Briefly, 5–6-week old mice were anesthetized by i.p. injection of a mixture of ketamine/xylazine (120 and 10 mg/kg respectively), positioned in a stereotaxic frame (David Kopf Instruments, Tujunga, CA), and intracerebrally injected (white matter of the fimbria fornix; Paxinos and Franklin Atlas coordinates: AP =  − 2.7, ML =  − 3.0 and DV =  − 4.0) with 0.5 μl of either monomeric or oligomeric [^125^I]Aβ derivatives (10 μM; flow rate: 0.1 μl/min) with the aid of a 10-μl Hamilton 701 RN syringe and a 30/2″/3S RN needle. To avoid reflux, the needle was left in position for two minutes after injection and then slowly withdrawn. One-hour post-injection, the mice under deep anesthesia were sacrificed by trans-cardiac perfusion with PBS (2 min, medium flow pump, Fisher Scientific 13-876-2, 10 ml/min flow rate) followed by brain tissue harvest. Radioactivity remaining in the whole brain was assessed in the Perkin Elmer 1470 γ-counter.

### Statistical analysis

Statistical significance of differences between two groups was assessed by unpaired Student’s *t*-test using Prism Graph Pad (San Diego, CA). Differences were considered statistically significant at values of *P* ≤ 0.05.

## Results

### Aβ immunohistochemistry

**Fig. 1:**
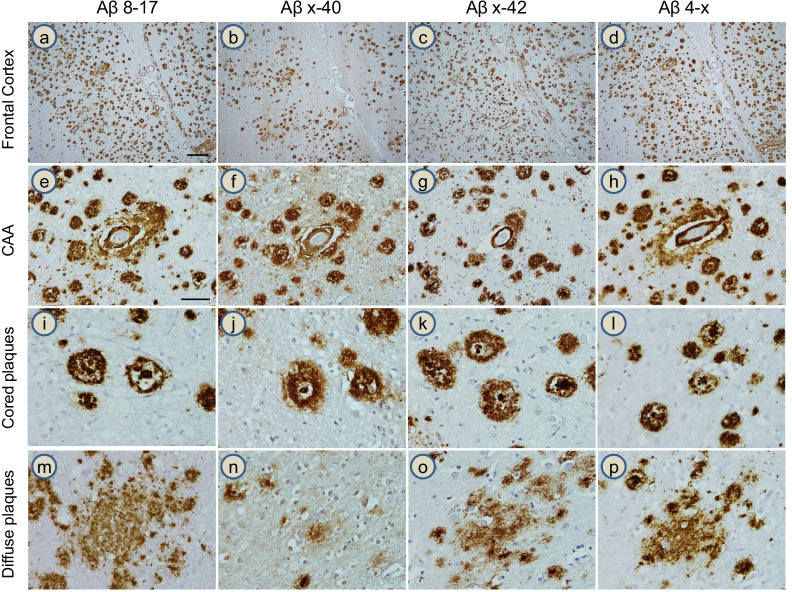
Aβ immunohistochemistry in AD frontal cortex. Four Aβ antibodies were used on sequential sections to investigate the presence of different Aβ proteoforms in AD pathological lesions. On low-magnification images (**a**–**d**), Aβ antibodies targeting positions Aβ8-17, Aβx-42, Aβ4-x, and Aβx-40 all stained plaques and CAA deposits, although the latter displayed lower immunoreactivity. At higher magnification, all Aβ peptides were observed in CAA (**e–h**) and in cored plaques (**i**–**l**). Diffuse plaques, however, showed an abundance of Aβ8-17, Aβx-42 and Aβ4-x reactivity (panels **m**, **o**, and **p**, respectively) with only sparse Aβx-40 staining (**n**). All images were acquired from Case 2. Scale bar in **a** represents 100 µm in (**a**–**d**); scale bar in **e** represents 50 µm (**e**–**p**)

The topographical distribution of the different Aβ proteoforms was evaluated using a combination of light and confocal fluorescence microscopy methodologies on sequential sections from the frontal cortex of cases previously diagnosed with AD (Table 1). Antibodies to Aβ8-17, Aβx-40, Aβx-42, and Aβ4-x recognizing different Aβ epitopes were used to determine the localization of the different peptides within the pathological lesions. As illustrated in Fig. 1, all types of Aβ deposits—parenchymal plaques, pre-amyloid lesions, and CAA—were stained by all of the antibodies tested, although the different peptide forms were present in different proportions. As illustrated in the low-magnification images of Case 2 (Fig. 1a–d), the abundance of Aβ4-x deposits matched that of the lesions containing the Aβ8-17 and Aβx-42 forms, whereas the proportion of Aβ plaques stained for Aβx-40 in the grey matter was lower than those highlighted by antibodies specific for the other peptides. Higher-magnification images showed presence of all species in CAA deposits (Fig. 1e–h) and cored plaques (Fig. 1i–l), although weaker Aβ4-x immunoreactivity was found (or observed) in the diffuse plaques (Fig. 1m–p).

**Fig. 2:**
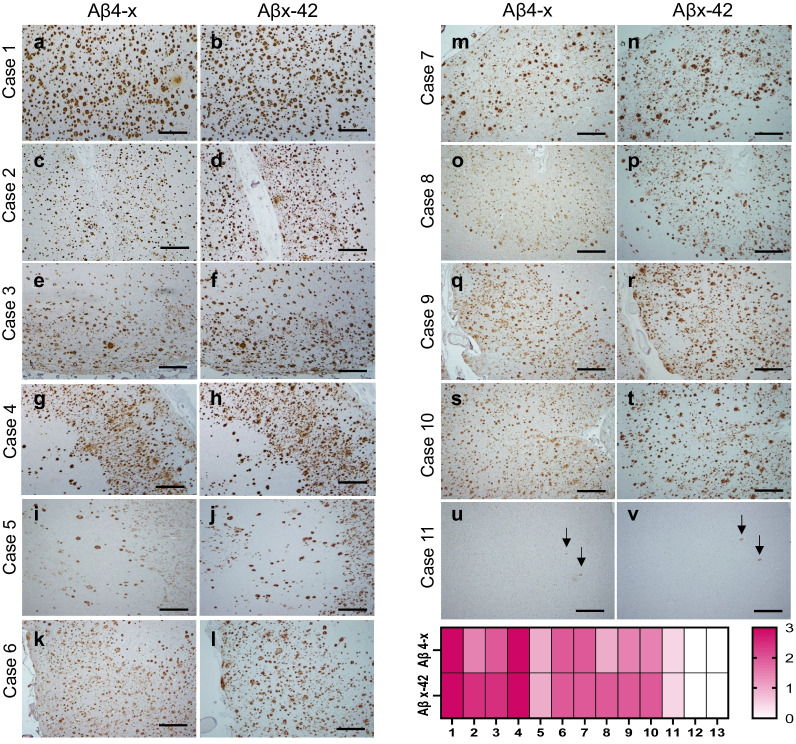
Aβ4-x and Aβx-42 immunohistochemical analyses in AD cases. Low-magnification images showing Aβ4-x and Aβx-42 immunohistochemical staining carried out on all cases listed in Table 1. Heat map illustrates the comparative semiquantitative analysis of the immunostainings encompassing all pathological hallmarks. No staining was detected in non-demented control cases 12 and 13. Scale bars, 500 µm

Additional low-magnification images of sequential sections of all the AD cases listed in Table 1 stained with antibodies specifically recognizing Aβ4-x and Aβx-42 further illustrate the abundance of Aβ species truncated at position 4, despite differences in disease onset, gender, disease duration, or apoE genotype among the cases (Fig. 2). Immunohistochemical staining of three non-demented controls (Cases 11–13) did not show any parenchymal staining with the antibodies, although in Case 11, CAA was barely highlighted by both Aβ4-x and Aβx-42 antibodies in the absence of Aβ plaques (Fig. 2u, v). The other two control cases (Cases 12 and 13) did not depict any detectable Aβ deposits (images not shown). The heat-map illustrates the semiquantitative analysis of the low-magnification immunostaining. Consistent with our previously reported evaluation in a different set of 20 AD patients [51], all AD cases in Table 1 showed Aβ4-x reactivity, and in about half of them Aβ4-x and Aβx-42 antibodies exhibited a comparable staining intensity. The three other cases showed less immunoreactivity for Aβ4-x than for Aβx-42. More in-depth neuropathological evaluations are needed to fully determine whether this variability relates to actual disease-associated differences in the progression, onset, or natural history of AD or simply reflects mere technical issues. In this sense, changes in tissue fixation, tissue thickness, pre-treatment, and/or slight modifications in reaction conditions with the different antibodies including uncontrollable variability in the room temperature conditions, among others, are known to affect the immunohistochemical procedures [75].

**Fig. 3:**
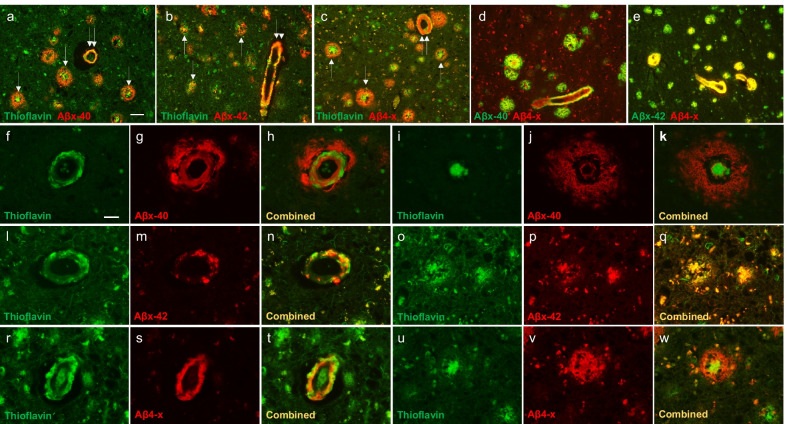
Aβ immunofluorescence combined with Thioflavin S staining. Low-magnification images of Aβx-40, Aβx-42, or Aβ4-x combined with Thioflavin (**a**–**c**) show the distribution of Aβ peptides tested and their overlap with the fibrillar amyloid components from Case 1. All peptides are present in amyloid fibrillar conformation in CAA (double white arrows) and in cored plaques (white arrows). Double immunofluorescence with Aβx-40/Aβ4-x and Aβx-42/Aβ4-x was carried out to illustrate the overlap of peptides starting at position 4 with those ending at position either 40 or 42 within the different pathological lesions from Case 1 (**d**–**e**). High power magnification images show the distribution of the Aβ peptides carrying the Aβx-40, Aβx-42, and Aβ4-x epitopes in CAA and cored plaques (**f**–**w**). Aβx-40 staining was observed in deposits with fibrillar amyloid conformations within the blood vessel, but also in Thioflavin-negative perivascular lesions (**f**–**h**, Case 2). Aβx-40 was also abundant in the cored plaques, both in the halo of the core and radiating around the core (**i**–**k**, Case 2) but seldomly in the central core itself. Aβx-42 was found deposited in CAA, in patchy deposits around the vessels, and in cored plaques (**l**–**q**, Case 2). Aβ4-x was present in the CAA and throughout the cored plaques, including the central core (**r**–**w**, Case 1). Scale bar, 50 µm in (**a**–**e**) and 20 µm in (**f**–**w**)

**Fig. 4:**
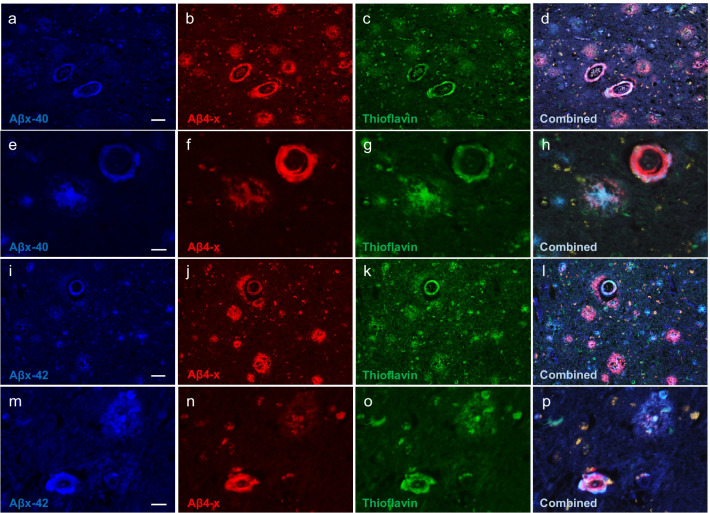
Double Aβ immunofluorescence analysis combined with Thioflavin staining. Detection of Aβ4-x epitopes in combination with either Aβx-40 or Aβx-42 was performed together with Thioflavin staining to highlight the presence of the respective Aβ peptides in the amyloid and pre-amyloid lesions, illustrated from Case 1. Both Aβx-40 and Aβ4-x proteoforms were present in CAA and parenchymal plaques (**a**–**h**). Higher-magnification images illustrate the co-localization of both peptide species in blood vessels and in cored plaques (**e**–**h**). A similar pattern is observed for Aβ4-x and Aβx-42 staining at lower and higher magnifications (**i**–**p**). Scale bars, 50 µm in (**a**–**d**) and (**i**–**l**), 20 µm in (**e**–**h**) and (**m**–**p**)

Combining single antibody immunofluorescence with Thioflavin S staining highlighted the distribution of the different epitopes in the lesions and their association with the fibrillar deposits (Fig. 3a–c, f–w), whereas double immunohistochemical analysis emphasized the overlap of Aβ4-x species with the Aβx-40 and Aβx-42 proteoforms in plaques and CAA (Fig. 3d, e). These colocalizations were further highlighted by combining double immunohistochemical analysis with Thioflavin S staining, which illustrated the preferential location of Aβ4-x species in CAA and cored plaques with fibrillar amyloid conformation (Fig. 4), strongly suggesting that these truncated species might have poor clearance characteristics as they are associated with the most insoluble part of the amyloid deposits. It should be noted that the slight differences observed between the staining patterns of the single immunohistochemical analysis using DAB as the chromogen and the double immunofluorescent analysis using Thioflavin counterstain, originated as an unavoidable technical issue. A high concentration of formic acid is known to quench the Thioflavin fluorescence as this stringent acid treatment breaks down the beta-pleated structures affecting the dye binding [76], a detrimental effect observed not only in paraffin-embedded sections—as in the current work—but also in frozen tissue sections subjected to this antigen retrieval condition [77]. Thus, to compatibilize the Thioflavin staining with the analysis of the Aβ antibodies, the concentration of the formic acid being used for pretreatment had to be reduced to 70% to maintain the in situ fibrillar amyloid conformation of the peptides and allow Thioflavin recognition. This weaker concentration of formic acid while maintaining the structural configuration of the deposits somewhat compromise the full immunohistochemical detection of the Aβ antigens, resulting in slight differences in staining intensity observed between the DAB chromogen illustrated in Fig. 1 and the immunofluorescence images shown in Fig. 3.

### Structural analyses of full-length and truncated Aβ peptides

**Fig. 5:**
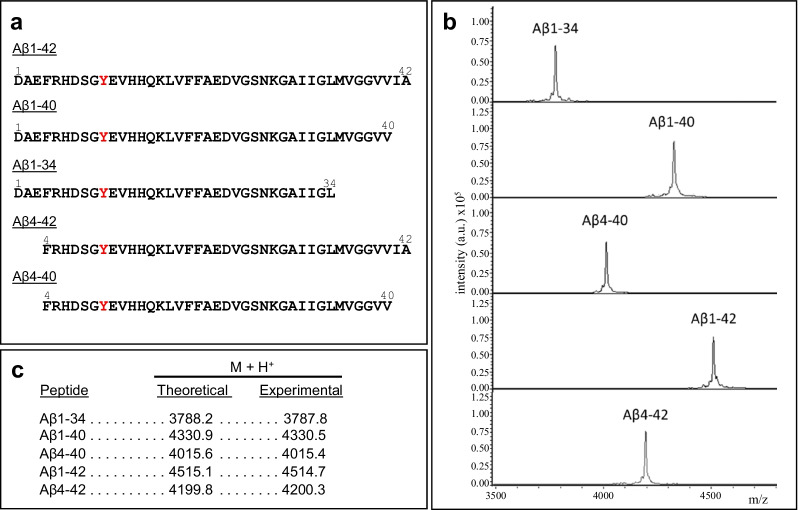
Full-length and truncated Aβ peptides employed in the evaluation of brain efflux. **a** Amino acid sequences in one letter code. Red font highlights the Tyr residue targeted by the radioiodination protocol. **b** MALDI-ToF MS profiles evaluated in a Bruker Daltonics Autoflex MALDI-TOF mass spectrometer illustrate the purity of the synthetic homologues. **c** Theoretical molecular masses of the different full-length and truncated Aβ peptides and the experimental masses determined by MS

**Fig. 6:**
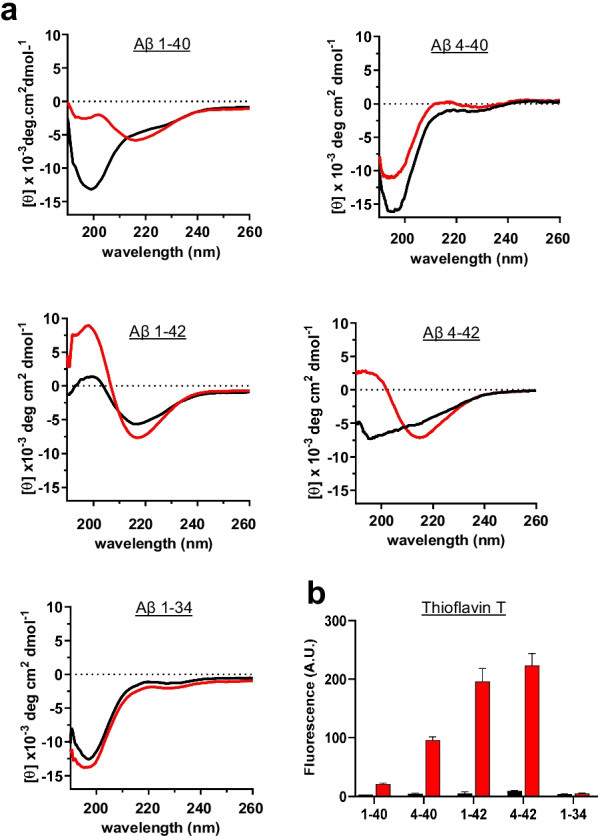
Biophysical and structural characterization of full-length and truncated Aβ peptides employed in the brain clearance experiments. **a** Circular Dichroism (CD) analysis to illustrate changes in secondary structure of HIFP-treated peptides freshly solubilized in 10 mM PO4 buffer containing 150 mM NaF (black lines) and the 24-h aggregated counterparts (red lines). In all cases, data represent the mean of 15 scans after subtraction of background readings of the respective buffer blanks. **b** Fluorescence evaluation of Thioflavin T binding to the respective synthetic homologues either freshly reconstituted (black bars) or after 24-h incubation under physiological salt concentrations (red bars). Results are expressed in arbitrary units (A.U.) and represent the mean ± SEM of three independent experiments after subtraction of blank levels

The purity and structural characteristics of the peptides tested in the in vivo brain clearance experiments described below were evaluated by a combination of MS, CD spectroscopy, thioflavin-T binding and electron microscopy. Figure 5 illustrates the amino acid sequences and purity of the peptides used in our experimental paradigms. All of them share a tyrosine residue at position 10 (Fig. 5a), an amino acid that was used as the target for radioiodination in the experiments described below. MALDI-TOF analysis of each of the HPLC-purified peptides (Fig. 5b) demonstrated a single component with experimental mass within ± 1 unit of mass of the corresponding theoretical values (Fig. 5c). The HFIP-pretreated synthetic homologues were solubilized in a buffer with physiologic pH and salt concentration and oligomerized following the procedures described in “Methods”. The structural changes induced by the peptide oligomerization were visualized by CD spectroscopy, thioflavin-T binding and electron microscopy after negative staining. All peptides except for Aβ1-42 adopted a typical unordered conformation upon solubilization, exhibiting a classic CD minimum at 198 nm characteristic of random conformations (Fig. 6a, black spectra). Under identical experimental conditions, the highly fibrillogenic Aβ1-42 showed a CD spectrum with a typical minimum at 218 nm, indicative of the presence of β-sheet-enriched secondary structure. Upon oligomerization, this type of secondary structure became more relevant for Aβ1-42 and was also the dominant conformation of the N-terminally truncated derivative Aβ4-42 (Fig. 6a, red spectra). Removal of the two C-terminal amino acids from Aβ1-42 resulted in an attenuation of the structural transitions, as illustrated by the intermediate mixture of β-sheet and unordered structures exhibited by Aβ1-40. Additional C-terminal truncation of the full-length Aβ1-40 peptide abolished the formation of β-sheet structures, as illustrated by the behavior of Aβ1-34 which maintained the same unordered conformation before and after the 1-day incubation oligomerization procedure (Fig. 6a). Truncation of the oligomeric full-length Aβ1-40 at the N-terminus that generated Aβ4-40 did not result in an enrichment of β-structures; on the contrary, the truncation favored the formation of predominant unstructured random conformations. However, the β-structure of the oligomeric Aβ1-42 was not substantially affected by the same N-terminal truncation at position 4, corroborating the importance of the two extra C-terminal amino acids in the formation and stabilization of predominantly β-structures.

Thioflavin T binding, a property largely associated with the presence of β-sheet structures and typically correlating with the existence of fibrillar and/or protofibrillar components [39, 69, 78], showed poor fluorescence signals in the freshly solubilized peptides but exhibited an array of different responses in the case of the oligomerized counterparts. The oligomerized peptides rich in β structures (Aβ1-42 and Aβ4-42) exhibited the highest thioflavin T binding whereas the C-terminally truncated Aβ1-34 that remained unordered throughout the experimental time frame showed a negligible fluorescence signal (Fig. 6b). Aβ1-40 and its N-terminal-truncated homologue Aβ4-40 exhibited low-to-intermediate thioflavin T binding activity. Interestingly, although the N-terminal truncation of Aβ1-40 generating Aβ4-40 did not result in an increased content of β-sheet structures in the latter, the truncated peptide exhibited higher thioflavin-binding activity than the full-length counterpart Aβ1-40, suggesting that other structural properties besides the β-sheet content play a role in the thioflavin-binding response.

**Fig. 7:**
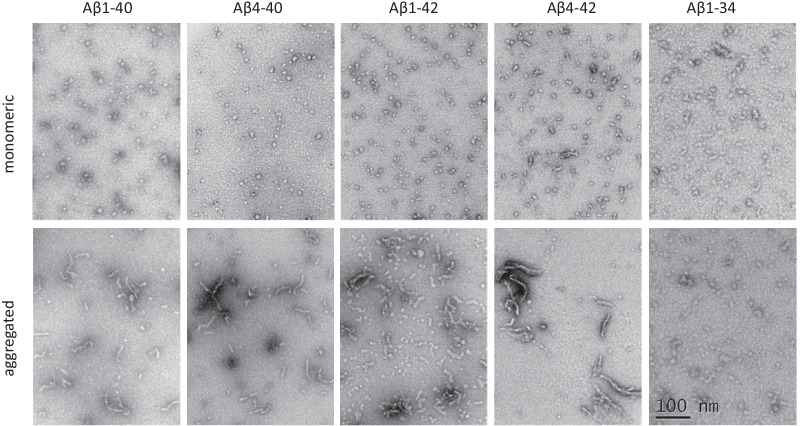
Structural assessment of full-length and truncated Aβ peptides by electron microscopy. Evaluation of the conformational assemblies exhibited by the different Aβ homologues employed in the intracerebral clearance experiments, both in their monomeric state and upon 24-h incubation/oligomerization. Scale bar, 100 nm

Final assessment of the conformational structures exhibited by the different peptide preparations employed in the current studies was conducted by electron microscopy analysis after negative staining with uranyl acetate. As indicated in Fig. 7, all monomeric peptide forms that exhibited almost negligible binding to thioflavin T showed the presence of very similar—if not identical—small globular structures of ≤ 10 nm in diameter. After the aggregation procedure, all peptides with the exception of Aβ1-34 showed the presence of oligomeric structures of < 100 nm as well as longer protofibrillar assemblies of different lengths, up to ~ 150 nm for Aβ4-42 but of shorter lengths for the rest of the peptides. Consistent with its unordered conformation and its poor fibrillogenic propensity evidenced by the absence of thioflavin T binding, Aβ1-34 did not form protofibrilar elements within our experimental time frame.

### Brain clearance of full-length and truncated Aβ peptides in monomeric and oligomeric conformations

**Fig. 8:**
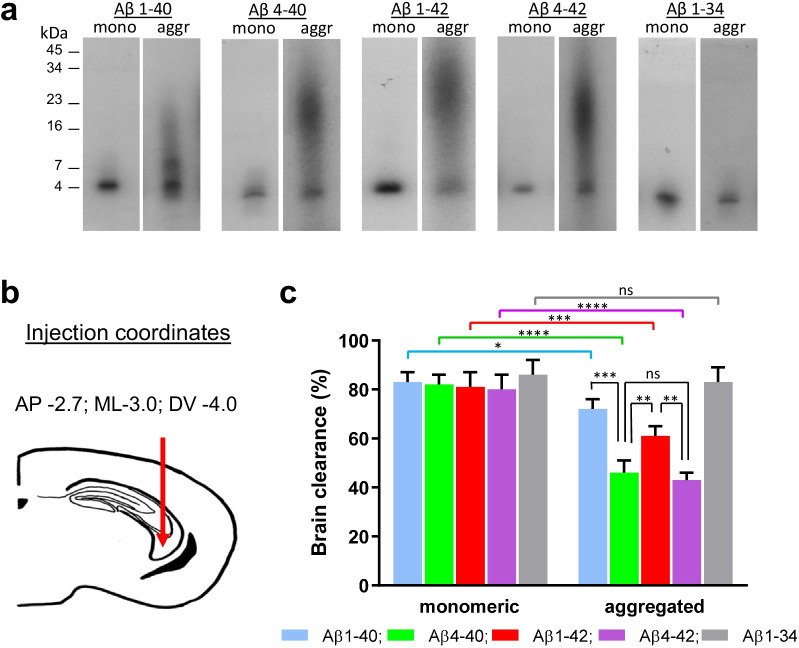
Intracerebral injection and brain clearance of full-length and truncated Aβ proteoforms. **a** Autoradiogram following electrophoretic separation of monomeric and 24-h-aggregated/oligomeric preparations of [^125^I]-labeled Aβ homologs on 16.5% SDS–polyacrylamide gels illustrates oligomerization profiles of the different Aβ forms prior to the intracerebral injection. **b** Schematic of the needle location for the intra-cerebral injection of the different Aβ proteoforms. **c** Brain clearance of radiolabeled full-length and truncated Aβ species in their monomeric conformations or after 24-h oligomerization. In all cases, bars illustrate percentage cleared relative to the total injected radioactivity; values represent mean ± SD obtained from inoculation of 5–9 mice (*t*-test, *P < 0.05; **P < 0.01; ***P < 0.001; ****P < 0.0001)

The different Aβ peptides in their respective monomeric and oligomeric conformations were labelled with Na[I^125^] at the tyrosine residue located at position 10 of the intact molecule (Fig. 5a), followed by removal of free iodine using a 1.8 kDa cut-off polyacrylamide desalting column, as described in Materials and Methods. As illustrated in Fig. 8a, autoradiography after SDS-gel electrophoresis confirmed the monomeric or the oligomeric composition of the respective preparations prior to their brain injection. As indicated in the pertinent autoradiograms, all monomeric preparations displayed a single band at ~ 4 kDa or slightly below in the case of the shorter peptide Aβ1-34, whereas the oligomeric preparations varied in size according to the aggregation propensity of the different peptides. The 24-h incubated Aβ1-40 showed presence of low-molecular-mass oligomeric species, exhibiting a mixture of SDS-resistant dimeric, trimeric and tetrameric assemblies together with a remnant of monomeric species. The more amyloidogenic Aβ1-42 as well as the N-terminal-truncated Aβ4-40 and Aβ4-42 exhibited the presence of SDS-resistant oligomers of larger molecular mass, from tetramers to dodecamers. It should be mentioned that the remnants of the ~ 4 kDa component observed in all oligomerized samples likely represent either non-oligomerized monomeric forms of the peptides or disaggregated products of non-SDS resistant aggregates.

The [^125^I]-labeled monomeric and oligomeric preparations of the full-length and truncated peptides illustrated in Fig. 8a were intra-cerebrally inoculated into young (5 to 6-week-old) C57BL/6 mice (Fig. 8b) to evaluate their respective efflux from brain. Figure 8c depicts the clearance of monomeric and oligomeric radiolabeled Aβ species from brain interstitial fluid—estimated based on the radioactivity remaining in the whole brain at 60 min post-injection—compared to the total injected radioactivity. Brain efflux of all monomeric Aβ species tested was fast, with ~ 80% cleared within the 60-min duration of the experiment. Although showing no significant difference in brain clearance compared to the other monomeric Aβ species, the C-terminally truncated Aβ1-34 consistently showed a trend for a higher brain efflux. Retention of oligomeric preparations was consistently higher than that of monomeric forms, with truncations at the N-terminus greatly enhancing brain retention and C-terminal truncations favoring brain elimination. The full-length oligomeric [^125^I]Aβ1-42 showed significantly less clearance compared to its monomeric counterpart (61% ± 4% *vs* 81% ± 6%, *P* < 0.001). Removal of two amino acids at the C-terminus of Aβ1-42 slightly increased the clearance of oligomeric [^125^I]Aβ1-40 (72% ± 4% *vs* 83% ± 6% for the monomeric counterpart, *P* < 0.05), whereas further removal of another additional four amino acids abrogated aggregation and deeply improved brain elimination. In this sense, [^125^I]Aβ1-34, incubated under the same experimental conditions as the other peptides, did not oligomerize within the 24-h experimental timeframe and therefore, clearance values were not significantly different when using fresh monomeric preparations of the peptide or 24 h-preincubated ones (83% ± 6% *vs* 86% ± 6%, respectively).

Brain retention of oligomeric forms of Aβ1-40 and Aβ1-42 was consistent with the dissimilar degree of aggregation exhibited by the full-length peptides prior to the intracerebral injections. The higher aggregation-prone Aβ1-42 was cleared from the brain less efficiently than Aβ1-40 (61% ± 4% *vs* 72% ± 4%; *P* < 0.01). N-terminal truncation at position 4 of the full-length Aβ1-42 further reduced its brain clearance. The oligomeric [^125^I]Aβ4-42 not only exhibited a significant reduction in brain elimination compared to its monomeric counterpart (43% ± 3% *vs* 80% ± 6%, *P* < 0.0001), but also showed enhanced retention when compared to the full-length oligomeric homologue [^125^I]Aβ1-42 (43% ± 3% for Aβ4-42 oligomers *vs* 61% ± 4% for Aβ1-42 oligomers, *P* < 0.01). Similar clearance values were obtained for the shorter truncated peptide [^125^I]Aβ4-40 (46% ± 5% for the oligomeric *vs* 82% ± 4% for the monomeric form, *P* < 0.0001). Highlighting the relevance of truncations at position 4, the retention of oligomeric preparations of [^125^I]Aβ4-40 also surpassed that of oligomerized [^125^I]Aβ1-40 (46% ± 5% *vs* 72% ± 4%, respectively, *P* < 0.001) as well as that of oligomeric [^125^I]Aβ1-42 (46% ± 5% *vs* 61% ± 4%, *P* < 0.01) and was comparable to that of oligomeric [^125^I]Aβ4-42 (46% ± 5% *vs* 43% ± 3%, n.s.).

## Discussion

Brain accumulation of Aβ is considered a central element in the pathophysiology of AD. Notably, despite innumerable studies addressing Aβ pathogenicity, the heterogeneity of Aβ species in brain tissue and CSF specimens—extending far beyond the canonical Aβ1-40/Aβ1-42 proteoforms—has only recently started to emerge [35, 39, 40, 44, 45, 79, 80]. The presence of N- and C-terminal-truncated species was initially reported as reflecting the presence of alternative cleavage sites for the APP processing enzymes BACE1—with capacity to generate species starting at Asp1 or Glu11—and γ-secretase—yielding peptides ending at residues 38, 40, and 42—as well as the activity of α-secretase, an enzyme associated with the non-amylogenic pathway, capable of forming the shorter peptides Aβ1-15 and Aβ1-16 [42, 81, 82]. Thereafter, many other truncated fragments have been identified in brain homogenates and biological fluids, likely reflecting the collective action of Aβ-degrading resident enzymes of the brain proteolytic machinery [83]. Using targeted-proteomic approaches, our previous work provided insight into the extent of Aβ heterogeneity in brain tissues and biological fluids from sporadic and familial forms of AD [39, 84–86], findings corroborated and in some cases expanded by others [45, 79, 80, 87]. The use of a multi-step biochemical retrieval approach that sequentially extracts water-soluble, detergent-soluble, and formic acid-soluble species, followed by peptide identification through immunoprecipitation and MS analysis, demonstrated the contrasting distribution of Aβ-truncated forms in biological fluids and within brain deposits, revealing a characteristic peptide profile in humans, non-human primates, and APP transgenic models [38, 39, 84]. Fragments exhibiting C-terminal truncations and therefore lacking hydrophobic regions of the molecule, are more easily extracted from brain deposits, and consistently retrieved in physiologic water-based buffers. Conversely, peptides with N-terminal truncation starting at Phe4 but exhibiting intact C-terminus, particularly Aβ4-42 which lacks the hydrophilic region while maintaining the mid-domain and the hydrophobic C-terminus, are more insoluble, thereby requiring the use of SDS or formic acid treatment for tissue extraction. In agreement with the properties suggested by their tissue extractability, the more soluble C-terminally cleaved species exhibit no toxicity [27], have a faster plasma turnover kinetics compared to the full-length Aβ1-40/Aβ1-42 counterparts [88], and are likely to be more easily eliminated from brain, as evidenced by their abundance in CSF [38, 39]. In contrast, N-terminal truncation at position 4 induces changes in the biophysical properties of the Aβ fragments, primarily affecting their solubility and imposing restrictions for efficient tissue retrieval and immunohistochemical detection. Biochemical/biophysical studies with synthetic homologues have confirmed the differential solubility and contrasting fibrillogenic characteristics of the truncated species, highlighting their high amyloidogenic propensity, and likely contribution to amyloid pathogenesis in agreement with previous work from our lab and other groups [39, 89].

The current work has taken advantage of the use of novel custom-generated monoclonal antibodies specific for Aβ forms starting at position-4 and blind for full-length species, in conjunction with antibodies recognizing peptides ending either at position 40, Aβx-40, or at position 42, Aβx-42. The use of this species-specific panel together with pan-Aβ antibodies in immunohistochemical analysis of AD brain specimens provided a clear assessment of the abundance of the different peptide forms in parenchymal and vascular lesions as well as of the differential localization of full-length and truncated species within the Aβ deposits. The data presented here highlight the broad distribution of the different Aβ species among the amyloid and pre-amyloid deposits, demonstrating the presence of all peptide forms investigated—including the N-terminally cleaved fragments Aβ4-x—in plaques and CAA deposits. Notably and consistent with previous findings from our lab as well as the work of other groups, species starting at Phe4 are abundantly distributed and represent a dominant fraction in vascular deposits and cored plaques overlapping in many cases with the more fibrillar areas of the thioflavin-S-positive lesions [39, 44, 90].

Over the last decade, increasing evidence indicates that defective clearance of Aβ protein from the brain is one of the main mechanisms leading to brain Aβ accumulation and a critical contributor to AD pathophysiology. The reduced brain elimination—particularly in the elderly—affects the delicate balance among degree of Aβ production, dynamics of aggregation, and rate of brain efflux. Glial phagocytosis, local enzymatic degradation, efflux through the vascular endothelium at the BBB, transport with the bulk flow of interstitial fluid into the CSF across the choroid plexus epithelium, peri- and para-vascular drainage along basement membranes in capillary and artery walls, and elimination via the more recently described glymphatic system and meningeal lymphatic vessels are among the mechanisms under current investigation and have demonstrated participation in brain Aβ removal [56, 58, 91–95]. Of all these pathways, clearance across the BBB is definitely the most studied and one of the most significant contributors to Aβ brain removal, accounting for ~ 75% of the overall Aβ efflux in humans [96]. Once cleared to the blood stream, consistent with its very low and steady concentration in plasma, the full-length Aβ has a very short half-life of ~ 3 min in mice, similar to that of the peptide hormone insulin or oxytocin [53]. Systemic Aβ catabolism occurs mainly in the liver through LRP1-mediated hepatocyte uptake and proteolytic degradation followed by secretion of the intact peptide together with its proteolytically-derived fragments into the bile [97]. Consistent with these findings, chronic liver diseases have been associated with an increased risk for developing AD, and impaired peripheral Aβ clearance has been reported as a characteristic feature of nonalcoholic fatty liver disease and is a common finding in patients with cirrhotic liver injury [98, 99].

The current study sought to understand how truncation of Aβ that generates pathophysiologically relevant species affects the brain efflux while correlating the clearance capability of the different proteoforms with their dissimilar aggregation/oligomerization properties. Consistent with the short half-life of Aβ in the periphery, the data presented in this work corroborate the fast rate of Aβ brain elimination and support the negative influence exerted by peptide oligomerization. Brain efflux of all monomeric Aβ species tested was fast, with ≥ 80% of the intracerebrally injected material cleared within 1 h. The retention of oligomeric forms of Aβ was, in all cases, consistently higher than that of the monomeric counterparts. Aβ1-34, which did not oligomerize within the current experimental window, showed no statistically significant difference in clearance between monomeric and 24-h aggregated preparations.

Truncations at the N-terminus—particularly those generating Aβ4-42, a fragment of relatively high abundance within the amyloid deposits and exhibiting a higher aggregation/oligomerization tendency—greatly enhanced brain retention, supporting the notion that the brain elimination of the different Aβ proteoforms decreases as their oligomerization tendency increases. It remains to be elucidated whether this decreased clearance simply reflects the inability of the structurally more-stable aggregates to maintain their solubility in interstitial fluid, thereby precluding their brain elimination, or whether it correlates with a decreased efficiency of the truncated forms to bind to the brain efflux transporters. In spite of the numerous studies on the mechanisms contributing to the brain removal of Aβ, no available information addresses the potential pathways participating in the elimination of truncated forms of the peptide. The recent discovery of N-terminally truncated forms in plasma, together with the demonstration of Aβ4-x as well as pyroglutamate-modified AβpE3-x and AβpE11-x in CSF, points out the ability of these forms to cross the blood–brain and the brain-CSF barriers [22, 100, 101].

The present data illustrating the enhanced brain retention of Aβ species exhibiting high aggregation tendency add to the current evidence supporting the pathogenic role of Aβ oligomerization in AD. Small intermediate soluble Aβ oligomers have been implicated in the increasingly severe synaptic dysfunction and neuronal loss, which lead to the progressive dementia associated in later stages of the disease with extensive Aβ pathology [4, 102–105]. Through a delayed brain removal, oligomeric species—particularly those constituted by N-terminal-truncated peptides that exhibit the highest brain retention—have higher potential to exert their pathogenic activity. Furthermore, since the process of multimerization is concentration-dependent, the persistence of oligomeric forms of Aβ within the brain is likely to contribute to the amyloidogenic loop, exacerbating the assembly of higher-molecular-mass species and/or recruiting soluble forms of Aβ into multimolecular complexes in a nucleation/seeding effect, which is increasingly recognized as a significant contributor to the pathogenesis of neurodegenerative disorders [106, 107].

It is interesting to note that the percentages of Aβ1-40 cleared from the brains of 5–6-week-old mice reported here—82% in the case of monomeric preparations and 62% for oligomeric forms—were considerably higher than those previously published by our lab after comparable injections in 26–30-week-old mice (60% for monomeric and < 40% for oligomeric forms) [38]. These differences suggest a detrimental role for aging in the brain elimination of the peptide. In this sense, it is important to emphasize that the cerebral microvasculature, a main player in the regulation of Aβ homeostasis, suffers important anatomical age-associated changes including decline in capillary numbers [108], increase in vessel tortuosity [109], and a significantly lower number of intracerebral vessels visualized by magnetic resonance angiography [110]. These changes coexist with pathological abnormalities encompassing age-related fibrosis, vessel wall thickening [111], and alterations in vascular basement membranes [94, 112], all of which undoubtedly interfere with normal vessel function including drainage along perivascular spaces [113, 114], a known contributor to brain Aβ elimination. These anatomical abnormalities, added to the age-associated decrease of the Aβ efflux receptors low density lipoprotein-related protein 1 and permeability glycoprotein at the choroid plexus [115, 116] and our previously reported decline in the expression of these transporters at the capillary level [117], not only in aged murine models but also in correlation with regional Aβ accumulation in AD brains [118], highlight the significance of age-associated changes for the mechanisms of brain Aβ elimination.

The complexity of the interlinked Aβ brain removal mechanisms, which encompass different cell populations and in some cases overlapping cellular pathways, and the challenges posed by the Aβ proteoform diversity, explain to a certain point the lack of success of therapeutic strategies targeting individual components of this multiplex cascade. A better understanding of the relevance of Aβ4-42 and other N-terminal-truncated fragments and a deeper knowledge of the mechanisms regulating their brain-blood-CSF homeostasis are paramount to provide insight into the role of Aβ heterogeneity in the complex mechanisms of Alzheimer's pathophysiology and open up new avenues for translational opportunities.

## Conclusions

Understanding the molecular basis that regulates the delicate balance among Aβ production, its dynamics of aggregation and rate of clearance is a crucial step towards identification of brain homeostatic mechanisms that modulate the undesirable formation of pathogenic oligomeric assemblies in AD. In spite of the extensive research, the contribution of Aβ heterogeneity and the presence of numerous N- and C-terminally truncated fragments that consistently populate the Aβ peptidome, their homeostatic regulation and potential contribution to disease pathogenesis remain to be elucidated. The data presented here highlight the importance of Aβ4-x species, which demonstrate high aggregation/oligomerization proclivity, are localized in the most insoluble part of the amyloid deposits in AD cases, and exhibit higher brain retention than full-length counterparts. A better understanding of the relevance of Aβ4-42 and other N-terminal-truncated fragments is needed to provide insight into the role of Aβ heterogeneity in the complex mechanisms of AD pathophysiology and identify new potential therapeutic targets.

## Data Availability

The datasets that support the findings of this study are available from the corresponding author upon reasonable request.

## Abbreviations

Aβ: Amyloid-β
AD: Alzheimer’s disease
BBB: Blood–brain barrier
CAA: Cerebral amyloid angiopathy
CD: Circular dichroism
CSF: Cerebrospinal fluid
DAB: 3,3′-Diaminobenzidine
EM: Electron microscopy
HFIP: Hexafluoro-isopropanol
MALDI-TOF: Matrix-assisted laser desorption ionization time-of-flight
MS: Mass spectrometry
PBS: Phosphate buffered saline
TBS-T: Tris-buffered saline containing 0.1% Tween 20
